# How is social support defined, categorized and measured in studies of work‐related musculoskeletal disorders among hospital nurses: A scoping review

**DOI:** 10.1111/jan.16356

**Published:** 2024-08-20

**Authors:** Enobong Gideon Asuquo, Sylvia Murphy‐Tighe, Ruth Ryan, Kieran O'Sullivan

**Affiliations:** ^1^ Department of Nursing and Midwifery University of Limerick Limerick Ireland; ^2^ Department of Nursing St. John's Hospital Limerick Limerick Ireland; ^3^ Health Research Institute University of Limerick Limerick Ireland; ^4^ School of Allied Health Faculty of Education and Health Sciences University of Limerick Limerick Ireland

**Keywords:** hospital, measurement, musculoskeletal disorders, nurse, nursing, pain, relationships, social support, well‐being, workplace

## Abstract

**Aim:**

To summarize current literature on the definition, categorization and measurement of social support in studies of work‐related musculoskeletal disorders among hospital nurses.

**Design:**

A scoping review.

**Data Sources:**

A literature search of four databases (CINAHL, Medline, Embase and Web of Science) was employed to map the evidence between January 2012 and April 2023 on the definition, categorization and measurement of social support in studies of work‐related musculoskeletal disorders among nurses in hospital settings.

**Review Methods:**

This review adopted Arksey and O'Malley's framework for conducting scoping reviews. Data extraction was reported using PRISMA Scoping Review guidelines and articles synthesized using a descriptive approach.

**Results:**

Fifteen studies met the criteria for inclusion in the review. Social support was distinctly defined in only two studies. Four main categories of social support identified were Co‐worker, Supervisor, Family and Overall support. Eight tools were found to measure social support, and the most used tool was the Job Content Questionnaire.

**Conclusions:**

The review identified that social support is often not explicitly defined. Furthermore, social support can be categorized and measured in different ways, using diverse tools. This variation may affect the understanding of social support and the approaches in measuring and providing social support in the workplace.

**Impact:**

Studies should clearly define the social support category evaluated, to facilitate comparisons between studies of nurses with work‐related musculoskeletal disorders. Healthcare managers should obtain feedback from nurses to ensure that the category of social support provided matches that which the nurse requires to ensure it helps the nurse from an organizational perspective. Healthy social relationships should be encouraged among nurses to promote nurses' well‐being and by extension patient care.

**Patient or Public Contribution:**

There was no patient or public involvement.

**What already is known**

- Work‐related musculoskeletal disorders are a leading cause of absenteeism and disability among nurses.
- Recent studies recommend exploring multidimensional approaches to prevent and reduce work‐related musculoskeletal disorders among nurses.
- Levels of social support among nurses can be linked to the prevalence of work‐related musculoskeletal disorders.

**What this paper adds**

- There was no standard definition of social support in the literature and that limits the understanding of social support in the workplace.
- Different, and sometimes overlapping categories of social support are reported.
- Studies should clearly determine, and define the social support category of interest to facilitate the choice of a suitable measurement tool.

**Implications for practice/policy**

- Identifying interventions that support the biopsychosocial needs of nurses such as social support may contribute to the reduction of work‐related musculoskeletal disorders.
- Understanding the different categories of social support may influence the choice and provision of social support for nurses, supervisors and workplace managers.
- A specific measurement tool for social support among nurses may aid proper evaluation of social support.

## 1 INTRODUCTION

Work‐related musculoskeletal disorders is a term used to describe a wide range of musculoskeletal complaints experienced in the workplace which affects joints, tendons, muscles and soft tissues (Yang et al., 2021). Work‐related musculoskeletal disorders are a significant occupational health problem in many professions (Asuquo et al., 2021; HSE‐Britain, 2022). A high prevalence of work‐related musculoskeletal disorders is reported in nursing, resulting in chronic pain, disability, absenteeism (Van Hoof et al., 2021) and poor quality of care (Yoshioka et al., 2018; Zhang et al., 2020).

Several factors have been linked to the development of work‐related musculoskeletal disorders such as physical strain at work, the work environment and psychosocial factors (Ellapen & Narsigan, 2014; Ouni et al., 2020). However, most preventive interventions have focused on physical factors such as manual handling training programmes, which have been found to be relatively ineffective as a sole treatment for work‐related musculoskeletal disorders (Van Hoof et al., 2018). Consequently, recent studies have suggested further investigation on the role of various psychosocial factors in the development of work‐related musculoskeletal disorders (Yang et al., 2021).

Psychosocial risk factors affect a persons' coping ability (Bernal et al., 2015) and includes workload, job control, shift work, effort‐reward imbalance, job dissatisfaction, inadequate staffing, depression, anxiety and social support (Sembajwe et al., 2013; Yang et al., 2021).

Social support is often used in a broad sense to describe the perception or availability of supportive resources in a relationship that might enhance health and well‐being. These resources can be emotional, informational, companionship and tangible or intangible (Dam et al., 2016). Sources of social support include relationships with work colleagues (e.g. supervisors and co‐workers), as well as relationships within the community and families (Jennings et al., 2016; Zhang et al., 2020).

Various theories have been used to explain the association between social support and health such as (i) stress and coping theory, (ii) relational regulation theory and (iii) lifespan theory (Vila, 2021). These theories explain how available or perceived social support exerts a direct, or buffering effect on workers' stress, personality traits and coping skills to produce positive health outcomes. Social support is considered to have a significant effect on health and suffering among women (Gebhardt et al., 2021), influence on pain (Choobineh et al., 2021), and impacts safety and wellness among healthcare workers (Jennings et al., 2016). As regards healthcare workers, social support also affects nurses from different perspectives, including completion of nursing training by students (Conner, 2015), nursing leadership (Van der Heijden et al., 2017), staff turnover and organizational culture (Kim et al., 2018) and beyond the workplace, affecting nurses' sleep patterns, quality of life and well‐being with the potential of exposure to work‐related musculoskeletal disorders (Sembajwe et al., 2013).

Notably, the relationship between social support and work‐related musculoskeletal disorders on nurses in the health system can be demanding, and if poorly addressed, can result in more strain on a sector experiencing a shortage of nurses globally (Mai & Kim, 2022). The need for social support has recently been given attention in some sectors such as mental health, among first‐time mothers regarding maternal efficacy and post‐natal depression (Leahy‐Warren et al., 2012), adolescent's health and older person's care, particularly during the COVID‐19 pandemic (Galanis et al., 2021).

Notwithstanding that social support is an acknowledged contributor to work‐related musculoskeletal disorders, the relationship between these factors requires further research and examination (Barzideh et al., 2014; Freimann et al., 2016). One challenge in understanding this relationship is the diverse methods used to evaluate social support in different settings (Jolly et al., 2021). Hospitals, for instance, have nurses as the largest professional group and the highest population of nurses in one setting. This cluster of nurses comprises varying levels of expertise and high skilled work demand that may result in exposure to occupational hazards such as work‐related musculoskeletal disorders (Lin et al., 2020). There have been reports of high rates of sick leave among hospital nurses than in other nursing settings (Ose et al., 2022; Rocha et al., 2019). Hospitals also play a key role in identifying and implementing best practices to enhance safety while serving as a major training centre for future healthcare professionals such as nurses (Tiwaken et al., 2015). It may be beneficial to examine content‐relevant approaches to musculoskeletal research in need of specific focus within the hospital in this review (Thacker, 2020). The purpose of this scoping review is to review the current literature on how social support is defined, categorized and measured in studies of work‐related musculoskeletal disorders specifically among hospital nurses. The outcome of this scoping review may reveal evidence gaps that will guide further studies on social support in relation to work‐related musculoskeletal disorders which may facilitate the prevention and management of work‐related musculoskeletal disorders, to enhance nurses' well‐being and their ability to provide optimal care.

## 2 METHODS

A scoping review protocol was registered with Open Science Framework (Asuquo et al., 2023) on 6th March 2023 prior to the commencement of the literature search. The Scoping Review Framework by Arksey and O'Malley (2005) guided the six‐stage review process which includes identifying the research question, identifying relevant studies, study selection, charting the data, collating, summarizing, reporting the results and consultation. The application of the consultation stage of this framework is optional and dependent on the research method (Arksey & O'Malley, 2005). Therefore, it was not applied in this scoping review. The use of this framework was enhanced by further insights into each stage by Levac et al. (2010) and Peters et al. (2020). This approach is broad and is expected to find diverse evidence relating to methodology and other findings that will shape further studies (Gonçalves et al., 2021). The Joanna Briggs Institute Scoping Review guidelines advise the use of the PCC (Population, Concept, Context) framework for search terms and was adopted for this scoping review (Pollock et al., 2021). The PCC framework is commonly used in nursing research to identify the main concepts in the review question that will facilitate each database search (Bettany‐Saltikov, 2016; Peters et al., 2020). The PCC elements for this review were defined as follows.

- Population‐ Nurses experiencing work‐related musculoskeletal pain.; this review focused on studies involving nurses, inclusive of staff nurses, clinical nurse managers, clinical nurse specialists and advanced nurse practitioners who provide direct patient care. Student nurses and other healthcare workers were excluded due to study manageability.
- Concept‐ Social support; diverse forms of social support which have been evaluated in the development of work‐related musculoskeletal disorders were investigated in this review.
- Context‐ Studies in a general hospital setting, for example, emergency unit and intensive care unit. Context further includes studies that have identified various approaches of describing, investigating and measuring social support quantitatively in studies of work‐related musculoskeletal disorders among nurses. In addition, studies conducted in a general hospital setting which involves at least two hospital wards in one or two locations are included to support generalization of findings.

### 2.1 Review questions

This scoping review examined how social support was evaluated in studies of hospital nurses with work‐related musculoskeletal disorders. The primary research questions are as follows:

1. How is social support defined and categorized in studies of work‐related musculoskeletal disorders among hospital nurses?
2. How is social support measured in studies of work‐related musculoskeletal disorders among hospital nurses?

### 2.2 Relevant studies

Peer‐reviewed primary studies of the past 11 years and 4 months (January 2012 to April 2023) formed the source for this review.

#### 2.2.1 Inclusion criteria

The criteria for included studies are as follows:

1. Nurses including specialist nurses, nurse managers or a heterogenous sample with nurses' data analysed separately.
2. Studies conducted within one or two hospital settings that can be referred to in other terms as an acute setting or hospital.
3. Quantitative studies only to identify specific quantitative tools used, to measure social support and to manage the scale of the review.
4. Studies that clearly investigate the association between social support and work‐related musculoskeletal disorders in single or multiple body parts.
5. Studies published in English language or with English language translation.

#### 2.2.2 Exclusion criteria

1. Studies involving other hospital staff such as nursing aides, administrative staff, student nurses and unspecified nurses' sample.
2. Studies conducted in non‐hospital settings including GP practice, daycare centres, nursing homes, community nursing settings and clinical demonstration rooms are not considered.
3. Qualitative studies and literature reviews.
4. Non‐peer‐reviewed studies.
5. Studies in one speciality nursing unit only or involving one speciality set, for example, geriatric unit only, midwives only and perioperative nurses only.

### 2.3 Study selection

Four databases were searched to generate studies on the link between social support and work‐related musculoskeletal disorders among hospital nurses: CINAHL, MEDLINE, Embase and Web of Science. Combinations of search terms, truncations and boolean terms were also used to enhance the generation of all possible studies. The format of the following search terms was used; (Pain OR Musculoskeletal pain OR Work‐related musculoskeletal disorder* OR injur*) AND (social support or family support or network or inclusion or isolation) AND (nur* or nurses or hospital nurse or healthcare professional). In addition, the reference list of included and related studies was manually searched for studies that may be relevant to the review. If the full‐text version of a study was not available, inter‐library loans were sought.

### 2.4 Charting the data, collating and summarizing

Studies generated from the database search, citation and hand search (5571) were imported into EndNote reference management software (version 20) and duplicates were removed. A data extraction tool (PRISMA scr) was used to report the selection of studies. Following the screening, and eligibility stages, 15 studies were included in the review. Data extracted included details about the study and information relevant to the research questions. The result of the search is displayed in a data extraction table (Table 1), which contains the following information: author, country, sample size, study aims, social support categories and social support measuring tools.

**TABLE 1:** Settings and social support characteristics in the eligible studies.

Author/year	Country	Study aim	Sample	Social support categories	Measurement tool
Barzideh et al. (2014)	Iran	To investigate job stress dimensions and examine their relationship to work‐related musculoskeletal disorders among nurses	385	Co‐worker support Supervisor support	Job content questionnaire
Choobineh et al. (2021)	Iran	To investigate the association between job stress dimensions and the prevalence of lower back pain among Iranian hospital nurses	495	Co‐worker support Supervisor support	Job content questionnaire
Petersen and Marziale (2017)	Brazil	To characterize the sociodemographic aspects, work capacity and stress of nursing workers affected by musculoskeletal disorders and to analyse the association between musculoskeletal comorbidities, capacity, stress and social support	52	Overall social support	Job stress scale
Sembajwe et al. (2013)	United States of America	To assess the relationship between psychosocial factors at work and multi‐site musculoskeletal pain among patient care workers	1065	Coworker support Supervisor support	Job content questionnaire
Zhang et al. (2020)	United States of America	To study the association between the comorbidity of work‐related musculoskeletal disorders, depression and working conditions	397	Overall social support	Job content questionnaire
Hoe et al. (2012)	Australia	To investigate the relationship between sociodemographic, individual and workplace factors and neck pain alone, shoulder pain alone and neck and shoulder pain among nurses working across three public hospitals	1111	Co‐worker support Supervisor support	Job content questionnaire
Mai and Kim (2022)	Vietnam	To determine the moderating effect of job resources in the relationship between job demands and work‐related musculoskeletal disorders among hospital nurses	225	Co‐worker support Supervisor support	Job content questionnaire
Jennings et al. (2016)	United States of America	To examine relationships between family supportive organization perceptions and health outcomes, as well as how those relationships may depend on work schedule and family differences	438	Family support	Allen's (2001) measure of family support perceptions
Niu et al. (2023)	China	To explore the prevalence of musculoskeletal pain and investigate how sleep and psychological factors influence musculoskeletal pain in nursing	230	Family support	Adapted standardized nordic musculoskeletal questionnaire
Ouni et al. (2020)	Tunisia	To evaluate the prevalence of musculoskeletal issues and to search for relationships with individual characteristics and work‐related risk factors among two public hospital nurses	310	Overall social support	Job content questionnaire
Sadeghian et al. (2015)	Iran	To explore possible risk factors for lower back pain	246	Co‐worker support Supervisor support	Cultural and psychosocial influences on the disability study questionnaire
Yan et al. (2018)	China	To analyse the correlated influential factors between work‐related musculoskeletal disorders and nursing practice environment and quality of life and social support	1973	Overall social support	Social support rating scale
Fujii et al. (2013)	Japan	To examine the association of neck and shoulder discomfort with somatization and work‐related factors	515	Co‐worker support Supervisor support	Cultural and psychosocial influences on the disability study questionnaire
Chang et al. (2020)	United States of America	To examine the association between support exchange imbalance and nurses' musculoskeletal disorders via anger	410	Co‐worker support	Social support questionnaire
Freimann et al. (2016)	Estonia	To report the prevalence of musculoskeletal pain among hospital nurses and to explore the associations of work‐related psychosocial factors and mental health problems with musculoskeletal pain	408	Co‐worker support Supervisor support	Copenhagen psychosocial questionnaire II

## 3 RESULTS

### 3.1 Search outcome

**FIGURE 1:**
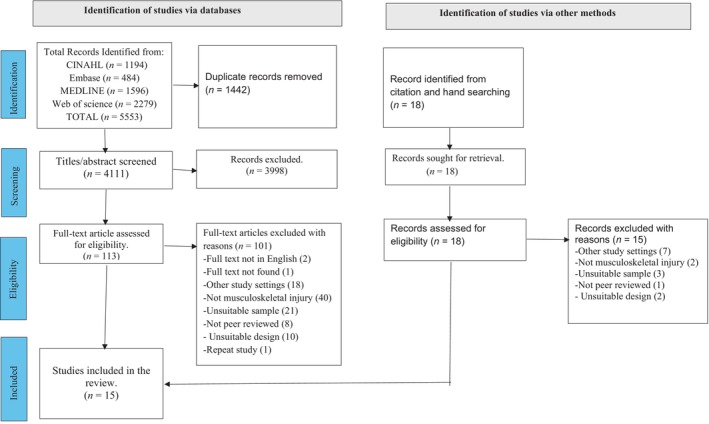
PRISMA‐ScR flowchart (Tricco et al., 2018). PRISMA‐ScR: Preferred Reporting Items for Systematic Reviews and Meta‐analyses for Scoping Reviews.

The initial search of the four databases generated 5553 studies. After the removal of duplicate studies through database automation and manually, 4111 articles remained. Following the title and abstract screening, a total of 3998 articles were excluded. Full‐text screening of the remaining 113 studies was performed with 101 articles being excluded, leaving 12 studies eligible for inclusion. A further 18 studies were retrieved through hand searching the reference list of the identified and related studies that were screened, resulting in 3 more eligible studies, making a total of 15 studies included in the review. The search result is reported in line with the Preferred Reporting Items for Systematic Reviews and Meta‐analyses for Scoping Reviews (PRISMA‐ScR), used together with the checklist (Tricco et al., 2018). The detail of the search is outlined in the PRISMA‐ScR flow chart (Figure 1).

### 3.2 Study and participant characteristics

The studies were conducted in 9 countries including some high‐income countries such as the United States of America and Australia. Five of the studies (Choobineh et al., 2021; Freimann et al., 2016; Fujii et al., 2013; Niu et al., 2023; Zhang et al., 2020) were conducted in one hospital setting, one conducted in a whole American region which covered all the acute hospitals in the area (Jennings et al., 2016). The research was undertaken in multi‐sites and five studies took place in two hospitals (Chang et al., 2020; Mai & Kim, 2022; Ouni et al., 2020; Petersen & Marziale, 2017; Sembajwe et al., 2013), two studies took place in three hospital locations (Hoe et al., 2012; Sadeghian et al., 2015), one covered 12 hospital settings (Yan et al., 2018) and one was conducted across 14 hospitals (Barzideh et al., 2014). Thirteen studies were cross‐sectional except two, (Jennings et al., 2016; Sadeghian et al., 2015) employed longitudinal designs.

The total population of the participants in the review was 8260. No sex mix was stated in a total of 1223 participants (Chang et al., 2020; Fujii et al., 2013; Petersen & Marziale, 2017; Sadeghian et al., 2015), while the remaining population were predominantly female (6915) and 122 males. The sample size of most studies was above 200 with one study having a nursing sample of under 100 (Barzideh et al., 2014). Out of the 15 included studies, eight involved participants with at least 1‐year of working duration in the study setting while (Chang et al., 2020; Hoe et al., 2012; Jennings et al., 2016; Sadeghian et al., 2015; Sembajwe et al., 2013; Zhang et al., 2020) did not specify the job period of the study participants.

In line with the general scoping review process, no quality appraisal assessment was done (Tricco et al., 2018), and all the studies that met the inclusion criteria were included in the review.

### 3.3 Research questions

#### 3.3.1 Definition and categorization of social support

Only two studies explicitly defined the concept of social support. Petersen and Marziale (2017) described social support as comprising of various interactions such as the relationships between co‐workers and supervisors, which are helpful for the smooth running of the workplace. In contrast, Chang et al. (2020) depicted social support as a range of helping behaviours that are performed by someone to help others. Although the other studies did not explicitly define social support, its meaning was implied in the measurements, result presentations and the discussion of findings. However, it should be noted that the aim of such studies was broad and not mainly to investigate social support.

It was difficult to categorize social support due to the research approaches employed in the studies. Some studies investigated social support generally (Ouni et al., 2020; Zhang et al., 2020) and one paper stated the categories investigated (Yan et al., 2018). Some studies investigated more than one category of social support. Overall, this review identified;

- Four main categories of social support which were (1) Co‐worker support, (2) Supervisor support, (3) Overall support and (4) Family support.
- Eight studies investigated co‐worker support and supervisor support together (Barzideh et al., 2014; Choobineh et al., 2021; Freimann et al., 2016; Fujii et al., 2013; Hoe et al., 2012; Mai & Kim, 2022; Sadeghian et al., 2015; Sembajwe et al., 2013).
- Four studies examined overall support only (Ouni et al., 2020; Petersen & Marziale, 2017; Yan et al., 2018; Zhang et al., 2020).
- Two papers assessed family support (Jennings et al., 2016; Niu et al., 2023).
- One study explored co‐worker support only (Chang et al., 2020).

Most studies considered social support primarily from an organizational perspective (Barzideh et al., 2014). Co‐worker support was generally described as the expectation or availability of assistance within the organization (Chang et al., 2020) but was not clearly explained in the papers. Likewise, all the papers that investigated supervisor support only referred to its implied meaning as the assistance provided by superiors at work. A unique perspective of this review was the overall support category which was presented as either a general support in the workplace (Ouni et al., 2020; Zhang et al., 2020) or as an umbrella term for other categories of social support identified in other papers (Petersen & Marziale, 2017; Zhang et al., 2020).

**FIGURE 2:**
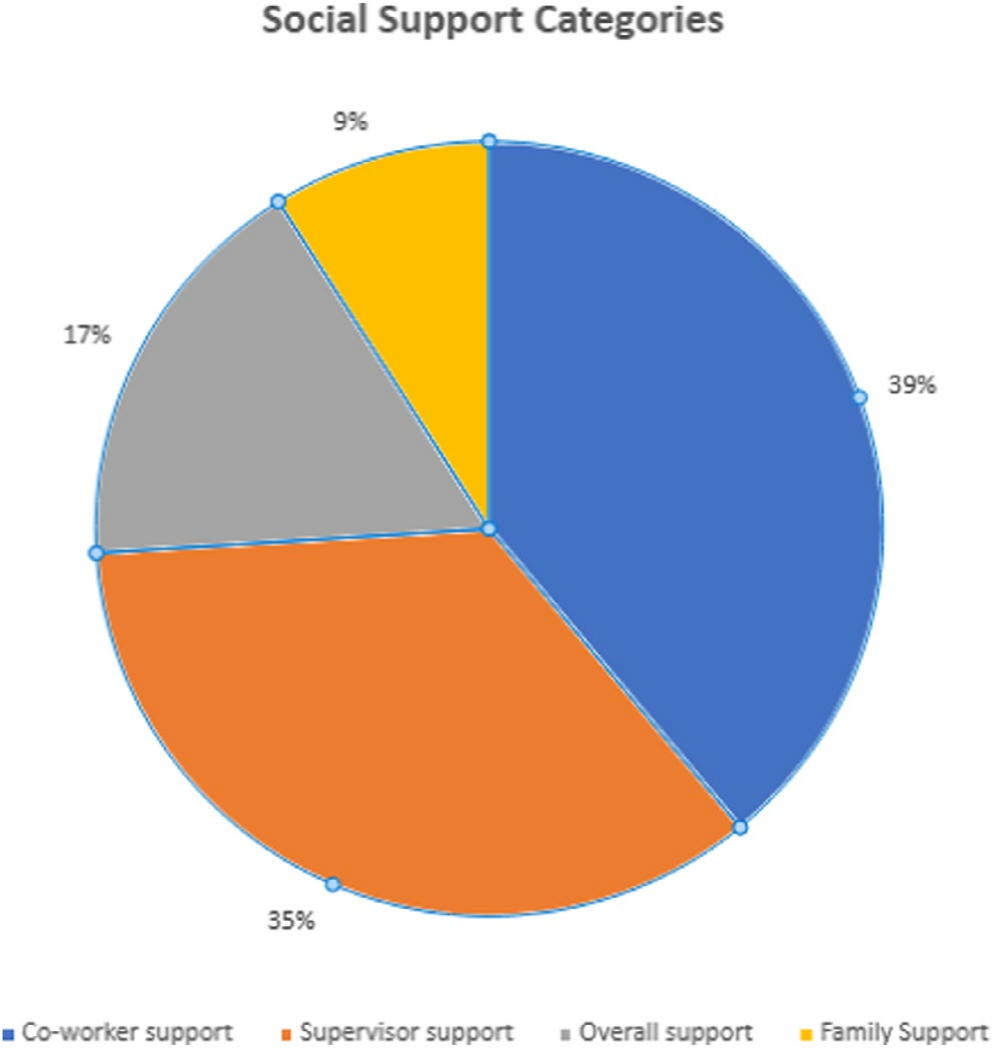
Four main social support categories.

As regards the family support category, it was similarly often lacking a detailed description of the concept. It was the least investigated category in the review. Notably, a study that explored family relationships reported on family support (Niu et al., 2023) while studies that examined relationships at work rather categorized social support as overall, co‐worker or supervisor support (Hoe et al., 2012; Sembajwe et al., 2013). A graphic illustration of the categories of social support in the review is presented in Figure 2. Co‐worker support was the most researched category of social support, being examined in nine studies (39%) (Barzideh et al., 2014; Chang et al., 2020; Choobineh et al., 2021; Freimann et al., 2016; Fujii et al., 2013; Hoe et al., 2012; Mai & Kim, 2022; Sadeghian et al., 2015; Sembajwe et al., 2013). Supervisor support was investigated in eight studies (35%) (Barzideh et al., 2014; Choobineh et al., 2021; Freimann et al., 2016; Fujii et al., 2013; Hoe et al., 2012; Mai & Kim, 2022; Sadeghian et al., 2015; Sembajwe et al., 2013), overall support (four studies, 17%) (Ouni et al., 2020; Petersen & Marziale, 2017; Yan et al., 2018; Zhang et al., 2020) and family support (two studies, 9%) (Jennings et al., 2016; Niu et al., 2023).

#### 3.3.2 Measurement of social support

The studies in this review used eight social support measurement tools.

- The most prominent tool used to measure social support was the ‘Job content questionnaire’ used in seven studies (Barzideh et al., 2014; Choobineh et al., 2021; Hoe et al., 2012; Mai & Kim, 2022; Ouni et al., 2020; Sembajwe et al., 2013; Zhang et al., 2020).
- The second most common tool was the ‘Cultural and Psychosocial Influences on Disability (CUPID) questionnaire’, adopted in two studies (Fujii et al., 2013; Sadeghian et al., 2015).

No other tool was used in more than one study. Notably, one tool could be used to measure different categories of social support. For example, the Job content questionnaire was used to measure co‐worker support, supervisor support and overall support but not family support. Similarly, both the CUPID (Fujii et al., 2013; Sadeghian et al., 2015) and Copenhagen questionnaires (Freimann et al., 2016) measured co‐worker and supervisor support, while the other measurement tools were used to measure only one category of social support (Table 1).

## 4 DISCUSSION

This scoping review synthesizes evidence on the definition, categorisation and measurement of social support. All 15 studies in the review investigated at least one category of social support and did so from different perspectives. Two studies defined social support and four main categories of social support were identified with some studies reporting more than one category of social support. The review outlined eight measurement tools that were used to measure social support. These tools were not specific to nursing groups and three were used to measure more than one category of social support.

### 4.1 Definition and categories of social support

#### 4.1.1 Defining social support

The two studies (Chang et al., 2020; Petersen & Marziale, 2017) which defined social support used slightly different language but are similar overall in how they defined social support. They both describe social support as ‘a connection between people within a particular setting’. This supports previous definitions of social support as a variety of interpersonal behaviours among workers that enhance psychological or behavioural functioning among individuals (Kogler Hill et al., 1989), in addition to ‘actions of others that are either helpful or intended to be helpful’ (Deelstra et al., 2003).

The lack of a detailed definition of social support in many studies may be connected to the studies focusing generally on psychosocial factors and only referring to social support as a subscale. Studies that aimed to investigate social support as a major factor among nurses tended to explore social support broadly (Chang et al., 2020; Petersen & Marziale, 2017) while studies that assessed social support as a component of other broad factors briefly explained social support, or did not discuss it at all. These different approaches to social support have been reported as a challenge in integrating social support research with health practices and further hinder understanding of the pathways through which social support relationships promote optimal well‐being (Feeney & Collins, 2015; Jolly et al., 2021). Therefore, it might be beneficial to clearly portray the social support construct of interest in a proposed study to enhance understanding of the topic and the choice of an appropriate research method to inform the generalization of findings and influence policies and practice.

#### 4.1.2 Categories of social support

The category of social support examined among the included studies appeared to be tailored towards the aim of the study. Social support was captured holistically as ‘overall support’ in the workplace and was described as the general relationship that exists among nurses in the hospital. Despite the discrepancy in the components of overall support, the review highlighted workplace relationships that influence stress at work which affects work‐related musculoskeletal disorders. However, the limited description of overall support could impede understanding of the support investigated, required and provided to nurses. This is in line with one of the proponents of overall support that highlights social support as available when there is a balance between required (subjective) support and provided (objective) support (Yan et al., 2018).

Co‐worker and supervisor support were the most prominent in the review. These were organizational and transactional. Generally, the support expected by each worker varies, relating to the tendency of individuals to compare themselves with internal and external standards (for example experience and other workers), and this could result in disproportionate levels of support. Workers with positive experiences in the workplace tend to experience high levels of support from co‐workers and supervisors which enhances positive outcomes (Chang et al., 2020; Yan et al., 2018). Previous studies have recommended further exploration of the different categories of social support through obtaining input from staff to ascertain their satisfaction with the support provided, compared with the support sought (Navajas‐Romero et al., 2020; Yamaguchi et al., 2016). Promoting support between co‐workers and supervisors can include promoting inclusive leadership styles and safe staffing ratios within the organization (Chevalier et al., 2021; Yamaguchi et al., 2016).

The category of family support did not evaluate support from the family, but rather how the organization provided support for employees to cope with family demands, including employees being able to detach themselves from personal problems and being willing to prioritize the organizational needs over the family (Niu et al., 2023). Since family support received limited focus in the review, more studies are needed on how family support could help employees cope, to help prevent the development and management of work‐related musculoskeletal disorders. Previous studies have advocated for better work‐life balance being essential for the reduction of musculoskeletal disorders and pain, through promoting job satisfaction and quality of life among nurses (Baur et al., 2018; Yamaguchi et al., 2016). Provision of support for nurses could be through modelling effective family management strategies, providing advice and developing hospital policies to help combine work with family life, especially in meeting the challenges of shift work (Barnett et al., 2019).

The terms used to categorize social support have been used interchangeably and without precision, with an overlap between the different categories. Other studies have identified career mentoring, task support, coaching and collegial support as categories of social support (Harris et al., 2007), older studies have attempted to categorize social support according to types and sources. Types include information support, material support or tangible support, appraisal support, emotional support and teaching (Birch, 1998; Kogler Hill et al., 1989). While sources include family, supervisor and co‐worker support (Baruch‐Feldman et al., 2002). This is in line with the findings of the review as different authors adopted different terms for social support categories in their study. Therefore, more clarity is required on the category investigated in related studies.

### 4.2 Measurement of social support

The review found eight different tools used to measure social support among nurses in the hospital environment. The most used measurement tool for quantifying social support in the research was the Job content questionnaire (Bernardes et al., 2014; Karasek, 2021). It appears to be preferred over the other seven tools identified in the review, probably due to its reported validity and the suitability for measuring social support specifically in different work settings (Bernal et al., 2015). However, a limitation to its use may be the requirement for approval from the copyright owner and the payment of a fee prior to its usage (Karasek, 2021). Although it measures co‐worker and supervisor support, other studies used the same question sets to measure overall support with the difference being the choice of the descriptive term for the category (Gottlieb & Bergen, 2010; Ouni et al., 2020). This was also applicable to the Copenhagen Psychosocial Questionnaire in terms of the categories measured (Freimann et al., 2016), but contrarily, the latter is available for use freely and without copyright permission and its validity has been well established (Burr et al., 2019; COPSOQ, 2020).

Within this review, some tools were adopted or modified for data collection, such as the Standardized Nordic Musculoskeletal Questionnaire which was one of only two questionnaires adapted and modified to measure family support (Niu et al., 2023). It is commonly used to measure physical symptoms of work‐related musculoskeletal disorders in body parts. Its validity and reliability were reported from a previous study (Bao et al., 2000). Also used to measure family support was the Allen 12‐items questionnaire. This tool is different in the review as it exclusively measured family support only (Jennings et al., 2016). It was designed to examine family perceptions of organizational support in relation to health outcomes (Allen, 2001). This tool was reported to be highly valid and reliable in measuring family support (Jennings et al., 2016). In related studies, the Allen questionnaire has been applied in studies of work‐life balance to obtain findings that will support nurses to satisfy family as well as work demands (Odle‐Dusseau et al., 2012; Seong, 2016).

The use of the different tools in work‐related musculoskeletal studies among nurses appears to follow well‐designed methods and the use of tools that were all reported to be valid and reliable in measuring social support. This provides valuable information on the understanding of social support among nurses (Chang et al., 2020; Fujii et al., 2013; Petersen & Marziale, 2017; Yan et al., 2018). Furthermore, the choice and application of measurement tools tend to be dependent on the study aims and researcher's perspective. In view of the overlap between different categories, caution is required in the categorization of social support in studies to enhance the choice of a suitable measurement tool.

## 5 LIMITATIONS

This scoping review was limited to studies published in English or with an English language translation to avoid linguistic and textual translation problems (Schwarz et al., 2016). The search was limited to studies published between January 2012 and April 2023. Included studies were limited to those conducted in hospital settings to provide a specific focus in this review.

The review team considered quality and comprehensiveness in capturing relevant literature and in choosing the most relevant databases, we acknowledge additional databases may have contributed to additional data. To overcome this limitation, and balance comprehensiveness and manageability of the review, our search was limited to four key databases. However, to maximize retrieval of relevant studies, this scoping review involved a search of reference lists and citations of included studies. In addition, no quality appraisal was conducted as this review focused on mapping evidence.

## 6 CLINICAL IMPLICATIONS

This review contributes further to the evidence base supporting the understanding of social support, how it is defined, categorized and measured which could support nurses in providing and receiving social support within the hospital. Implementation of the findings of this review on social support to work and training approaches can contribute to the prevention and treatment of work‐related musculoskeletal disorders. The provision of social support to nurses can be enhanced through developing family‐oriented policies to help combine work with family demands which may have a positive effect on work responsibilities and result in better care (Barnett et al., 2019). Co‐worker and supervisor support can equally be enhanced by promoting inclusive leadership styles and staffing within the hospital (Chevalier et al., 2021; Yamaguchi et al., 2016). Studies have equally recommended supportive social interventions such as increased job control and social bonding to reduce musculoskeletal symptoms and enhance nurses' well‐being (Habibi et al., 2012; Shojaei et al., 2019).

## 7 RESEARCH IMPLICATIONS

In the review, some regions had low representation, for example, Europe and Africa had one study each that met our inclusion criteria. This may indicate the need for further studies of social support and work‐related musculoskeletal disorders among nurses in these regions. Future reviews may include studies covering a span of more years than this review to map more evidence on this topic. None of the review studies investigated social support alone in relation to work‐related musculoskeletal disorders and thus the level of social support available to nurses within the hospital still appears understudied and vague which may affect nurses' well‐being. Further research should aim at investigating social support as a main variable with the potential to determine the balance between support and its link to work‐related musculoskeletal disorders among hospital nurses. A comparative study using different social support measuring tools could be conducted to assess the accuracy of the tools in measuring social support among nurses. Future studies may consider developing a specific measurement tool to assess social support for nurses as the measurement tools in this review were designed for the general workforce.

Qualitative methods could be employed to explore the lived experiences of nurses in providing and receiving support in the hospitals which could enhance insights and understanding of approaches for the provision of support for family demands that may in turn promote stability in family and workplace.

## 8 CONCLUSIONS

This review investigated how social support was defined, categorized and measured in studies of work‐related musculoskeletal disorders among nurses. Fifteen studies met the inclusion criteria for the review. Only two studies clearly defined social support. Four main categories of social support—co‐worker support, supervisor support, family support and overall support were identified. Different tools were used to measure various categories of social support. The most common tool used was the job content questionnaire. Identifying and highlighting interventions that enhance the health of nurses through better social support may contribute to the reduction and prevention of work‐related musculoskeletal disorders and improved well‐being. Understanding the different categories of social support may influence the choice and provision of social support for nurses, supervisors and workplace managers. It is important that nurses determine how, and if, the social support provided matches what is required to help nurses. Future studies should clearly define the social support category being evaluated, to facilitate the choice and comparison of a suitable measurement tool and to better explore how social support is associated with work‐related musculoskeletal disorders.

## AUTHOR CONTRIBUTIONS

E.G.A., S.M.T., R.R., K.O.S: Made substantial contributions to conception and design, or acquisition of data, or analysis and interpretation of data. E.G.A.: Involved in drafting the manuscript. S.M.T., R.R., K.O.S.: Revised the manuscript critically for important intellectual content. E.G.A., S.M.T., R.R., K.O.S.: Given final approval of the version to be published. E.G.A., S.M.T., R.R., K.O.S.: Agreed to be accountable for all aspects of the work in ensuring that questions related to the accuracy or integrity of any part of the work are appropriately investigated and resolved.

## FUNDING INFORMATION

This research received no specific grant from any funding agency in the public, commercial or not‐for‐profit sectors.

## CONFLICT OF INTEREST STATEMENT

None declared.

### PEER REVIEW

The peer review history for this article is available at https://www.webofscience.com/api/gateway/wos/peer‐review/10.1111/jan.16356.

## Data Availability

Data sharing is not applicable to this article as no new data were created or analyzed in this study.
